# A Two-Pathway Mathematical Model of the LH Response to GnRH that Predicts Self-Priming

**DOI:** 10.1155/2013/410348

**Published:** 2013-11-11

**Authors:** J. J. Evans, T. M. Wilkinson, D. J. N. Wall

**Affiliations:** ^1^Centre for Neuroendocrinology, University of Otago, Christchurch, New Zealand; ^2^MacDiarmid Institute for Advanced Materials and Nanoengineering, University of Otago, Christchurch, New Zealand; ^3^Department of Obstetrics and Gynaecology, University of Otago, Christchurch, New Zealand; ^4^Biomathematics Research Centre, University of Canterbury, Christchurch, New Zealand; ^5^Department of Mathematics, University of Canterbury, Christchurch, New Zealand

## Abstract

An acute response of LH to a stimulatory pulse of GnRH is modelled as a result of a pathway (Pathway I) that consists of two compartments including a single (rate limiting) intermediate. In addition, a second pathway (Pathway II) was added, consisting of an intermediate transcription factor and subsequently a synthesised protein. Pathway II had a delayed effect on LH release due to the time taken to produce the intermediate protein. The model included synergism between these two pathways, which yielded an augmented response. The model accounts for a number of observations, including GnRH self-priming and the biphasic pattern of LH response. The same model was used to fit the data of the LH response when gonadotrophs responded to the addition of oxytocin in the response with a shoulder on the profile. Pathway I is able to be conceptualised as the basic Ca^2+^-mediated pathway. Pathway II contains features characteristic of the cAMP-mediated pathway. Thus, we have provided an explanation for details of the nature of the profile of LH secretion and additionally enabled incorporation of cAMP in an integrating model. The study investigated the possibility of two interacting pathways being at the basis of both the shoulder on the LH surges and self-priming, and the model illustrates that this appears to be highly likely.

## 1. Introduction

 The release of luteinising hormone (LH) from gonadotrophs is central to reproductive function. LH exhibits an episodic pattern, which is a result of gonadotropin-releasing hormone (GnRH) being transported from the hypothalamus to the pituitary in pulses. Following GnRH occupation of its cognate receptor on gonadotrophs, its stimulatory signal is transduced to the cell utilising associated intracellular pathways [1] and secretion of luteinising hormone (LH) occurs. In an oestrogenised environment, a process called GnRH self-priming occurs, whereby an initial pulse of GnRH primes the gonadotrophs in readiness for a subsequent pulse of GnRH. The complexity is such that it is impossible to establish the detailed components of a stimulatory pulse of GnRH with simple reductionist concepts. Some laboratories have begun to construct mathematical models of the processes that are involved. We document here a model that applies to the female gonadotrophs in the preovulatory, oestrogenised state.

We produced a set of detailed data of the response in vitro, and for the model, we took note of the characteristics of the response: (i) a shoulder is present in the declining phase, (ii) enhancement occurs in a primed pulse, (iii) the enhancement is delayed; that is, by definition it did not occur at the initial pulse. We developed a model in which there are two pathways, Pathway I, which elicits a rapid LH response, and Pathway II, which has an effect that is delayed. The model required that the pathways interact and synergise.

We conceptualised a biological mechanism that was consistent with the model. The Ca^2+^-mediated pathway may be considered to be represented by Pathway I in our model. Our model is also consistent with additional effects being mediated by cyclic AMP, in whuch cAMP-mediated processes were noted to have characteristics of Pathway II. We then conceptualised the process of self-priming as being due to crosstalk between the Ca^2+^-mediated pathway and the cAMP/PKA pathway.

We produced a distilled model which can be interpreted as incorporating parallel effects of GnRH on LH release (Ca^2+^-stimulated exocytosis) and protein synthesis (cAMP transcription/translation). Oxytocin enhances the LH response to GnRH [2] and the model accommodated this effect.

## 2. Methods

### 2.1. Experimental

 To take into account the dynamic nature of the LH response, our model is based primarily on data from perifusion studies [2] previously reported. Hemipituitaries from single adult female rats at pro-oestrus were prepared and cut into two pieces. A divided hemipituitary was placed in a chamber of a perifusion apparatus [3] and medium (Medium 199) containing oestradiol that passed over the tissue for 220 minutes at a flow rate of 0.8 ml/min. At this time, a 4-minute pulse of GnRH (10 nM) was delivered. To investigate the effects of oxytocin (100 nM), the peptide was included continuously from 120 min prior to the first pulse of GnRH. Fractions were collected at 2 min or 5 min intervals and the eluate was assayed for LH by RIA. A second pulse of GnRH delivered after a 60-minute interval revealed GnRH 2 self-priming. 

### 2.2. Mathematical Model

**Figure 1:**
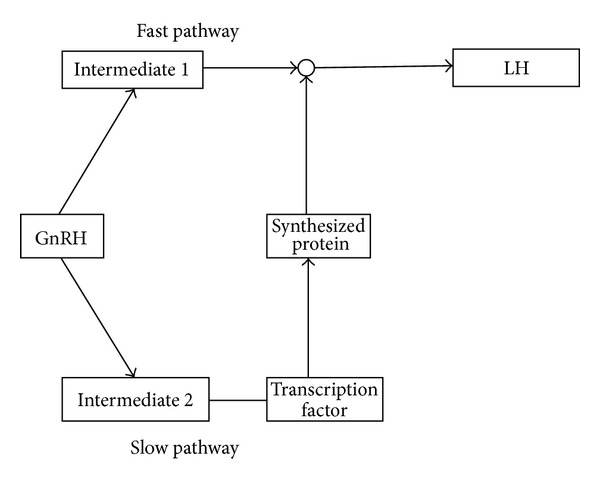
The elements of the interaction model. GnRH activates two pathways in this model, which synergize to produce an augmented LH response.

 A schematic diagram of our model is shown in Figure 1. The slow pathway (Pathway II) augments the fast pathway (Pathway I) by synergizing on the first intermediate, producing the priming effect, and is incorporated mathematically in Section 2.3.3.

Only those salient parameters that are essential and consistent with the model's ability to describe the experimental results are included. The introduction of more intermediates and thence parameters would make no difference to the model.

### 2.3. Model Parameters

*a* is the constant in Pathway I related to the strength of the responses of the intermediate and LH to GnRH. *p* is the time constant in Pathway I is related to the decrease in concentration of the intermediate compound. *q* is the time constant in Pathway I is related to the decrease in concentration of the GnRH. *m* is the constant in Pathway II related to the strength of the response of the intermediate and LH to GnRH. *r* is the time constant in Pathway II is related to the decrease in concentration of the intermediate compound. *T*_*i*_, *i* ∈ {1,2, 3} is the time parameter is related to the arrival of the multiple GnRH pulses; *i* = 1 corresponds to the arrival time of the first GnRH pulse. *t*_*p*_ is the time parameter is related to the delay in Pathway II. *T*_*P*_ is the absolute time parameter is related to the delay for the first pulse in Pathway II.

#### 2.3.1. Model for the LH Response by Pathway I

 The release of LH by Pathway I is described by two compartments. The first comprises the processes from GnRH stimulation to formation of an intermediate, and the second compartment results in the release of LH. An instantaneous impulsive input of GnRH is assumed; it is straightforward to account for a finite duration of input of GnRH. The rate of production of LH can be assumed to depend on a rate limiting step that produces the intermediate, *i*_1_(*t*), which we assume has a rapid rise and a time-dependent decline in concentration, with time being denoted by *t*. Any given intermediate before this step can be assumed to instantaneously increase to some peak concentration following binding of GnRH to its receptors. The differential equation modelling this is

(1) $$\begin{array}{c} \frac{di_{1}}{dt}\left(t\right)=a\left[\text{GnRH}\left(t\right)-pi_{1}\left(t\right)\right],\;t>T_{0} \end{array}$$

for the basal value *i*_1_(*T*_0_) = 0. We define

(2) $$\begin{array}{c} f\left(t\right)=ae^{-pt}H\left(t\right), \end{array}$$

where *H*(*t*) denotes the Heaviside unit step function

(3) $$\begin{array}{c} H\left(t\right)=\{\begin{array}{cc} 0, & t\leq 0\\ 1, & t>0. \end{array} \end{array}$$

It then follows that the solution to (1) is

(4) $$\begin{array}{c} i_{1}\left(t\right)=f\left(t-T_{0}\right)=ae^{-p(t-T_{0})}H\left(t-T_{0}\right). \end{array}$$

With the assumption that basal value of LH is [*LH*⁡](0) = 0, then LH will increase at a rate proportional to *i*_1_ and will decrease at a rate proportional to the LH concentration, as the more molecules of LH that are present, the greater the amount that will be consumed by cellular processes in a given time, and thus its equation is

(5) $$\begin{array}{c} \frac{d\left[\mathrm{LH}\right]}{dt}\left(t\right)=i_{1}\left(t\right)-q\left[\mathrm{LH}\right]\left(t\right),\;t>0. \end{array}$$

**Figure 2:**
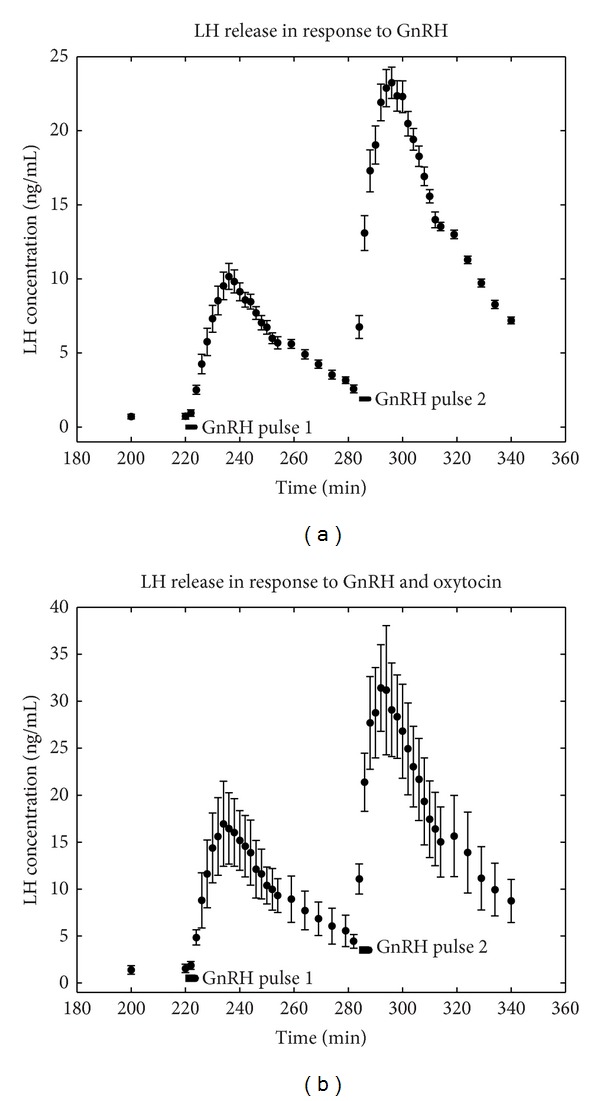
The LH response to two pulses of GnRH by halved hemipituitaries illustrating GnRH self-priming. The black bar shows the onset and duration of the GnRH pulse. (a) LH response to two GnRH pulses. (b) LH response to two GnRH pulses and continuous presence of oxytocin.

The time parameter *T*_0_ can be thought of as either the time at which the GnRH arrives at the cells (see Figure 2(a) where it appears to be around 220 minutes) or the time at which intermediate 1 is synthesised; it does not matter for the model which it is.

In practice as the concentration of GnRH increases, there is a maximum level of response that the LH secretory process can attain, which may be called saturation of the response. Therefore, the response of LH to GnRH is in general nonlinear. A saturation nonlinearity for the response to the GnRH input may then be incorporated into the model. However, it appears that nonlinearity does not substantially affect the basic shape of the intermediate response to GnRH [4]. Therefore, a linear model will suffice in this context, although the effect of the nonlinearity may be important to consider in the very early stages of the response. We note that nonlinearity in the response to the intermediate compound may also be incorporated in the model.

The model for Pathway I may also be extended to contain three or more compartments with the inclusion of additional intermediates. However, for the purposes of this study, a two-compartment model is satisfactory as we examine the longer term behaviour of the response to GnRH rather than the short-to-medium term responses considered by other authors [4–7].

#### 2.3.2. Model for the LH Response by Pathway II

 The kinetics of this pathway, once initiated, is assumed to be similar to that of Pathway I. Pathway II will involve steps that cause a delay at which LH is available, in particular, the transfer of the signal through transcription factors and protein synthesis. This means that effectively (for purposes of the model) it takes longer to initiate a detectable LH response, so a time lag, *t*_*p*_, is factored in. There is an indirect component by which augmentation of the Pathway I occurs. Pathway II does not directly produce LH release, so the levels of protein synthesis product, PT, are modelled instead. Thus [PT](*t*) will represent the temporal variation of the concentration of the protein stage prior to formation of LH through pathway II.

The intermediate *i*_2_(*t*) associated with the second pathway satisfies a similar but different equation to (1), here with the time constant *r*. However, in order to produce the time lag in PT, we assume that the second pathway activates the protein synthesis at an absolute time *t* = *T*_*P*_, *T*_*P*_ > *T*_0_, with relative delay *t*_*p*_ = *T*_*P*_ − *T*_0_. This has no effect on the model as the essential feature is that PT acts after time *t*_*p*_. Therefore, the temporal variation of *i*_2_ given that its GnRH response is *m* becomes

(6) $$\begin{array}{c} i_{2}\left(t\right)=me^{-r(t-T_{P})}H\left(t-T_{P}\right). \end{array}$$

So from Pathway II diagram, we see that the synthesised protein satisfies

(7) $$\begin{array}{c} \frac{d\left[\text{PT}\right]}{dt}\left(t\right)=i_{2}\left(t\right)-s\left[\text{PT}\right]\left(t\right),\;t>0, \end{array}$$

with [PT](0) = 0. So the PT response is

(8) $$\begin{array}{c} \left[\text{PT}\right]\left(t\right)=h\left(t-\left[T_{0}+t_{p}\right]\right) \end{array}$$

(9) $$\begin{array}{c} h\left(t\right)=\frac{m}{r-s}\left(e^{-s(t-T_{P})}-e^{-r(t-T_{P})}\right)H\left(t-\left[T_{0}+t_{p}\right]\right). \end{array}$$

For *r* ≠ *s*. As this occurs with probability 1 we do not quote the result for *r* = *s* in (9). It should be observed from (9), with *T*_*P*_ > *T*_0_ (or equivalently *t*_*p*_ = *T*_*P*_ − *T*_0_ > 0), that there is no output of protein until after *T*_*P*_. This implies that the positioning of intermediates or modelling the transcription factor will not affect our result. The important point is that PT is delayed by *T*_*P*_ which is a central time constant in the model. We use the analytic solution (9) as it simplifies our computational solutions in Section 3.1.

We note the model for Pathway I does not account for the presence of a shoulder in LH concentrations seen in the perifusion data. This is also the case when there is a nonlinearity in the response to GnRH or alternatively an additional compartment is incorporated in the model. Therefore, we modelled an interaction between Pathway I and the second pathway, Pathway II, that will produce both the effects of the shoulder and priming; this is done in Section 2.3.3. Therefore, the model includes a delay *T*_*P*_ caused by the time for production of the intermediate, PT. Biologically, the model is consistent with a protein being synthesised via Pathway II that has a synergistic interaction with Pathway I.

#### 2.3.3. Model with Interaction of the Two Pathways

To simulate the experimental results, it is necessary to achieve a model that combines the pathways. To provide the priming and synergistic effects seen in the experiments, it is appropriate for the slow pathway to augment the fast pathway for the first intermediate. A combined model is formulated for one LH surge by augmenting the response of the intermediate on Pathway I at a level proportional to the concentration of the intermediate protein of Pathway II. The combined pathway model is then

(10) $$\begin{array}{c} \frac{d\left[\mathrm{LH}\right]}{dt}\left(t\right)=\left(1+\frac{1}{a}\left[\text{PT}\right]\left(t\right)\right)i_{1}\left(t\right)-q\left[\mathrm{LH}\right]\left(t\right)\;t>0. \end{array}$$

It is not known where the interaction between the two pathways occurs with regard to the rate-limiting step of Pathway I. If the interaction is before the rate-limiting step, then the level of Pathway I intermediate will increase in proportion to the amount of protein. If it is after, the rate of production of the intermediate after the point of this interaction will increase in the same way as it would have if the interaction was earlier, as the augmentation will propagate down the pathway. Hence, the endpoint (LH secretion) will have the same result. We observe that our model now provides the behavioural features of gonadotropic LH surge incorporating both the shoulder and the self-priming effect (which are discussed further in Section 3) as the same mathematical modulation process, namely, the enhancement of the intermediate *i*_1_ through the delayed protein synthesis product [PT].

#### 2.3.4. Full Model with Multiple LH Surges

 When multiple GnRH surges occur, say *N* subsequent surges following the one at *T*_0_, the LH model is as given by (10), but the intermediate *i*_1_ and the synthesised protein PT are given, respectively, by

(11) $$\begin{array}{c} i_{1}\left(t\right)=\overset{N}{\underset{i=1}{\sum }}f\left(t-T_{i}\right), \end{array}$$

(12) $$\begin{array}{c} \left[\text{PT}\right]\left(t\right)=\overset{N}{\underset{i=1}{\sum }}h\left(t-\left[T_{i}+t_{p}\right]\right), \end{array}$$

where the subsequent GnRH surges occur at times *T*_*i*_, *i* ∈ {1,2,…, *N*}. 

#### 2.3.5. Combined Pathways Model with Oxytocin

The model was extended further to include the effect of exposure to oxytocin. To model the effect of oxytocin, a new parameter (OT) was added to the model, representing both the concentration of oxytocin (present from 0 and then constant over time) and a coefficient relating this to an effect on LH secretion. The model for LH surges is hence modified to

(13) d[LH⁡]dt(t)=(1+1a[[OT]+[PT](t)])  i1(t)−q[LH⁡](t) t>0,

and the intermediate *i*_1_ and the synthesised protein PT are given in (12). This model also accepts the inclusion of oxytocin to further modulate the intermediate *i*_1_.

## 3. Results

 The raw data [2], shown in Figure 2, includes both the initial LH secretory response to GnRH and also a second, primed response to a subsequent pulse of GnRH. The LH perifusate collections were made every 2 minutes after the beginning of the GnRH pulse (Figure 2(a) representing the average of 6 individually measured data sets). The error bars are the standard deviation of each data point across these 6 trials. The data in the presence of oxytocin are shown in Figure 2(b). It should be observed that the scales in Figure 2(b) are roughly twice those of Figure 2(a), illustrating the augmentation by oxytocin. 

### 3.1. Fitting the Model

 The mathematical model described in Methods is discussed in Section 2. The model was adapted to include the primed response. An optimisation programme was run in MATLAB, using nonlinear least-squares regression analysis to find the optimal values for the parameters, given the data. The least squares fit was weighted by the experimental standard deviation.

**Figure 3:**
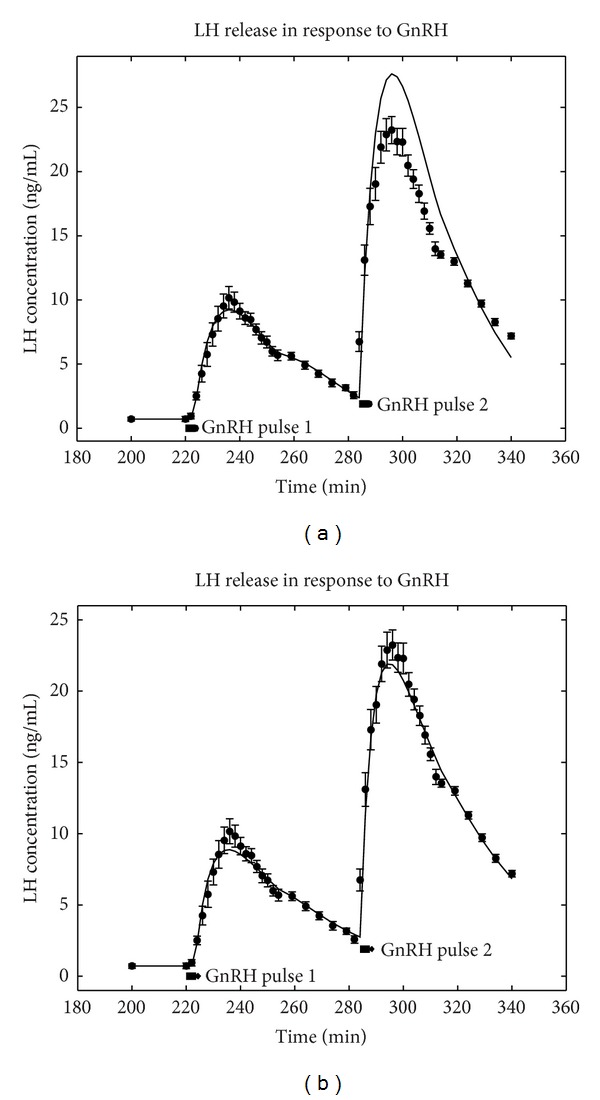
The optimal solution to the interaction model, (10), which results in an augmented response to the second stimulating pulse of GnRH. (a) Optimal solution to the first GnRH pulse with a prediction for the priming effect of the second stimulating pulse of GnRH. (b) In contrast showing what the model can achieve with an optimal solution to both GnRH pulses.

Firstly, numerical integration of (10) was used to optimise for the first LH surge only. We observe that this linear equation can be solved analytically, but for greater generality, our computer program used numerical integration. However, the analytic solution obtained for PT in (9) was used to avoid having to perform interpolation. It was necessary to constrain the parameters to positive values only, as negative values make no sense in the context of the biological situation. To fit the experimental basal LH value, an initial value of LH concentration [*LH*⁡](0) = *L*_0_ = 0.7178 ng/mL was set. The final parameter values obtained were *a* = 1.8001, *p* = 0.0846, *q* = 0.0641, *m* = 1.0480, *r* = 0.1501, *s* = 0.0178, *T*_0_ = 223.0, and *T*_*P*_ = 252.0 after the model output was optimised for the 25 LH values measured from the first pulse in Figure 2(a). These results are seen graphically (full line) in Figure 3(a) superimposed on the measured data producing an excellent fit for the first pulse with shoulder. Also seen are the results of the prediction when a second GnRH pulse is given with these same parameter values. It is seen that the model fits the primed response to the second LH surge in Figure 3(a) well with less than 20% overprediction in the peak. The fit is good for the first LH surge (*R* ^2^ = 0.981) and its extrapolation for the second pulse is promising in that the shape and size of the second LH surge have been approximated with reasonable and, in many cases, adequate accuracy. 

To illustrate that the model would fit the two surge data better, the model was adapted to allow for both surges to be considered. This was done by optimising the parameters from equation (10) from the data for both surges, with the second surge to occur at time *T*_0_ + 60. So numerical integration of (10) was used to optimise the parameters for the two LH surges. This is shown in Figure 3(b) and here there was no need to constrain the parameters to positive values, and the obtained optimal values were *a* = 1.98351, *p* = 0.159051, *q* = 0.0309806, *m* = 17.3815, *r* = 0.800823, *s* = 0.0557808, *T*_0_ = 223.111, and *T*_*P*_ = 254. This fit is good (*R* ^2^ = 0.982), although the shoulder in the data for the second LH surge is not replicated exactly by the model. However, the points around the shoulder were effectively fitted. We observe that the delay parameters *T*_0_ and *T*_*P*_ are not specified but predicted (fitted) by the model from the data in both cases. It should be pointed out that in this second run, the same number of parameters (10) were determined but there were approximately twice the number of data points (46). 

### 3.2. In the Presence of Oxytocin

**Figure 4:**
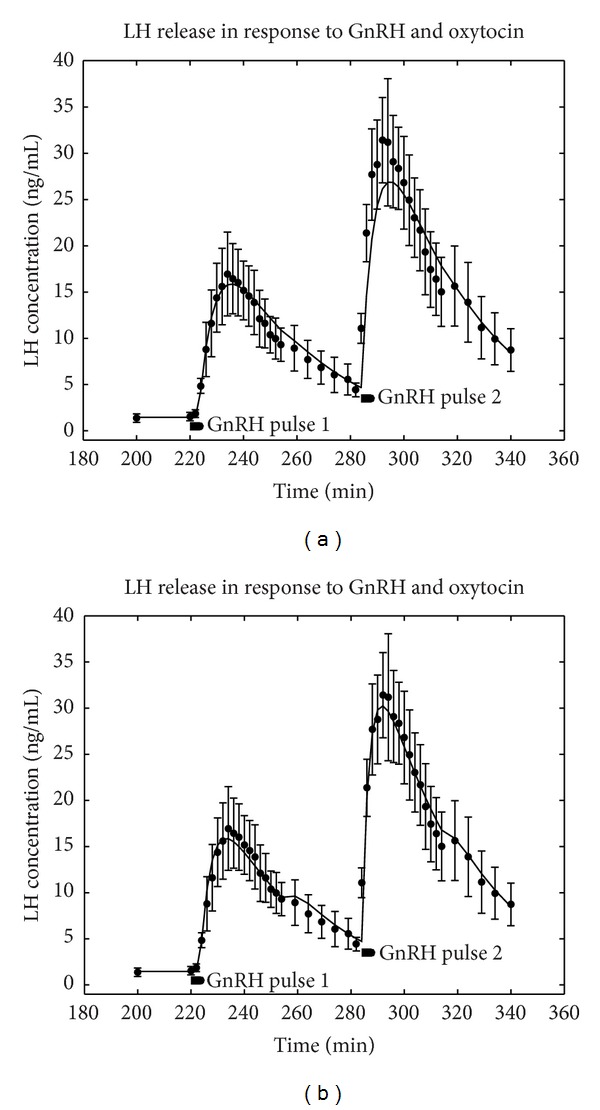
The optimal solution to the interaction model, (13), which results in an augmented response to the second stimulating pulse of GnRH. (a) The predicted result when the optimal solution for the OT parameter only was used. (b) In contrast showing what the model can achieve with an optimal solution for all parameters.

 A new initial value of LH concentration [*LH*⁡](0) = 1.4655 ng/mL is used, as the basal secretion is higher in the presence of oxytocin. An optimisation was carried out, with numerical integration of (13), to find the optimal value of the OT parameter only, assuming that the other parameters were unchanged from the optimal values obtained for GnRH only. This returned a value of (*a* + [OT]) = 3.51638 (the use of nonlinear least-squares regression can only find the total value of (*a* + [OT]) and not a and [OT] individually). This is a predicted result in that only the OT parameter has been chosen for the given data and the result is shown in Figure 4(a) (full line) superimposed on the measured data.

We next illustrate how well the model could fit the two surge data when the oxytocin data was used to optimise over all parameters; then (*a* + [OT]) = 4.50872, *p* = 0.218373, *q* = 0.0321599, *m* = 424.194, *r* = *s* = 0.234962, *T*_0_ = 223.183, and *T*_*P*_ = 254. The fit is good (*R* ^2^ = 0.975) and the result is shown in Figure 4(b) (full line). But the model prediction as shown in Figure 4(a), when only the OT parameter is fitted, is not significantly worse. Again it should be observed that the scales in Figure 4 are roughly twice those of Figure 3, so illustrating the model has reproduced the augmenting effect of oxytocin well.

Taking the optimal parameter values obtained across the data sets for GnRH only and GnRH plus oxytocin produces (*a* + [OT])/*a* = 4.50872/1.98351 = 2.2731 and performing a similar calculation with the basal secretion values produces 1.4655/0.7178 = 2.0417. The closeness of the two numbers 2.2731 and 2.0417 obtained by these two calculations suggests that the augmentation of the basal secretion by oxytocin is of a similar magnitude to the augmentation of the GnRH-stimulated surges. 

### 3.3. Parameter Sensitivity

#### 3.3.1. Pseudo-Random Gaussian Noise

To test the validity of the optimal values found from our computer programme pseudo-random Gaussian noise was added to the simulated LH points predicted by the model with these optimal parameter values, using the Monte Carlo method. By repeating this 10 times, 10 new datasets were obtained and for each, the model was optimised to obtain 10 new sets of parameters. For GnRH only, the parameter values converged to the true values as the hypothetical experimental error was reduced. 96 per cent of the 95% confidence intervals contained the true value. This result supports the validity of the optimal parameter values obtained. For GnRH plus oxytocin 92 percent of the 95% confidence intervals contained the true value. This again supports the validity of the optimal parameter values obtained. 

#### 3.3.2. Nonindependent Variation

To test the possibility that nonindependent variation of parameters occurred in the experimental data, parameters for each of the six individual experimental datasets were optimised and average and SDs of the values were determined. The mean parameter values obtained from optimal values for individual datasets were compared to the optimal parameter values obtained from the mean of the data. All of the 95% confidence intervals around these mean parameter values included the optimal value. When the set of parameters for GnRH only was compared to that for GnRH plus oxytocin, the only significant difference was associated with the presence of oxytocin. Thus, the results of the model reflected the proposed biological mechanisms. 

#### 3.3.3. Input of Constant GnRH

 It is also possible to examine the input of constant GnRH over an extended period in the model. Indeed a biphasic response similar to that inherent in our model is observed in experiments [8]. The biphasic response to constant GnRH input has previously been linked to the self-priming mechanism in an experimental context [8]. The production of a shoulder in LH production in response to a pulse of GnRH occurs after a similar time lag as the second phase of the biphasic response to the input of constant GnRH. Thus, it would appear that the three phenomena described here are interrelated. 

### 3.4. Biological Conceptualisation

 We can conceptualise Pathway I representing a rapid Ca^2+^ pathway and Pathway II being a pathway involving cAMP. Processes involving the cAMP pathway have been observed to have a progressive development over time. The delay in the effect of cAMP in inducing a visible change in LH secretion is consistent with the model as a result of the time taken for additional processes required for development of the response. The sequence of events in the cAMP-mediated pathway includes activation of PKA, its translocation to the nucleus, induction of a transcription factor such as cAMP response element binding protein (CREB) [9], and then transcription and translation steps. In particular, the rate-limiting step for the phosphorylation of CREB and cAMP-dependent transcription is translocation of PKA to the nucleus [10, 11]. The model requires augmentation of LH release by the cAMP pathway, as a result of synergistic interaction between the synthesised protein and the primary Ca^2+^ pathway. Nonlinearity in the response to either GnRH or the intermediate compound may be included in the model. Saturation nonlinearities for the production of adenyl cyclase and cAMP have been considered in other contexts [12]. Delays have been previously modelled, but for the Ca^2+^ pathway alone [13, 14].

### 3.5. The Model's Predictive Capability

The model was used to see how it provided predictive capability and it was found that when the model was fitted to the first LH surge only, it was able to predict the second surge with good accuracy (see Figure 3(a)).

The effect of oxytocin was such that the same model was able to be used and the mechanism predicted an increase in the value of parameter *a*. A very good fit to the data was produced.

With oxytocin exposure, there was an increase in the value of *a*, statistically significant at the level of 95% confidence, as predicted. Also, as predicted none of the other parameters displayed a similar statistically significant change in the presence of oxytocin.

**Figure 5:**
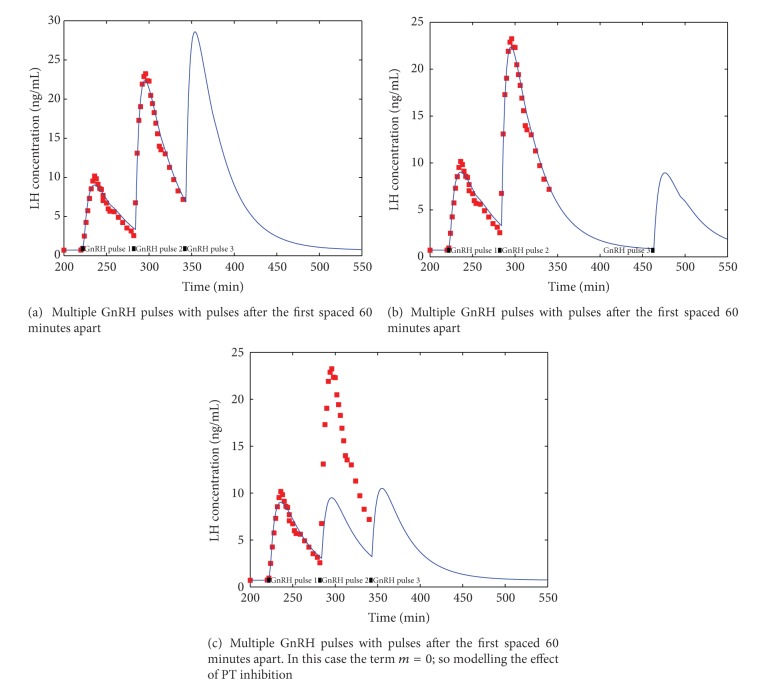
Multiple GnRH pulses simulation using the optimal parameters of Figure 3(b); also, the experimental data for the first two pulses (dotted lines) is shown.

 To further illustrate the predictive ability of our model, we show in Figure 5, using the optimal parameters of Figure 3(b), several changed scenarios for the model.  Figure 5(a) illustrates the self-priming enabled by our model on the third LH surge when a third GnRH pulse is presented to the system.  Figure 5(b) shows that when a third GnRH pulse is presented to the system after 180 minutes, the self-priming is minimal. These figures should be compared to the experimental data presented in Figures 1-2 of reference [15] illustrating experimental self-priming of GnRH. However, care should be taken with this comparison as the previous researchers [15] used a much longer pulse duration (10 min), and this will almost certainly have intracellular effects. When this comparison is done that it shows our model provides experimental prediction at the level expected.

Finally, in Figure 5(c), we illustrate the effect of inhibition of protein synthesis; this is modelled by setting *m* = 0 which has the effect of shutting down the synthesis of PT. No selfpriming is evident in the resulting LH pulses and neither are any shoulders present.This figure should be compared to the experimental data presented in Figure 3 of [16], where cycloheximide is used as a protein synthesis inhibitor, and it was shown that the self-priming effect was abolished.

### 3.6. Further Applications of the Model

 Such observations as those in the previous section suggest further potential to investigate other peptides that also interact with GnRH and which enhance GnRH-stimulated LH secretion, for example, pituitary adenylate cyclase-activating polypeptide (PACAP). PACAP stimulates cAMP expression with a time course in which the cAMP peaks at approximately 30 min [17, 18].

It would be possible to also apply the model to the LH profile resulting from the response to two pulses of GnRH produced in the presence of gonadotrophin surge attenuating/inhibiting factor (GnSAF) [19, 20]. Our model, in fact, predicts the pattern of secretion observed in a perifusion study (Figure 2) [21].

## 4. Discussion

 Our modelling has provided a system that explains a number of diverse aspects of the LH response: (1) GnRH self-priming; (2) a delayed effect following exposure to GnRH; (3) a shoulder on the declining phase of the response [2]; (4) potential understanding of the dynamic contribution of other peptides, such as PACAP and GnSAF, and implications for understanding and treating a number of reproductive pathologies and assisted technologies [22–24]; (5) the interactive effects of oxytocin on GnRH stimulation.

In the presence of oxytocin, it was shown that there was an increase in the value of *a*, statistically significant at the level of 95% confidence, as predicted. Also, as predicted none of the other parameters displayed a similar statistically significant change in the presence of oxytocin. It appeared therefore that oxytocin augments basal secretion to a similar degree as that which it augments secretion occurring in response to Pathway I,and oxytocin does not invoke Pathway II.The example illustrates that the model is able to be extended to characterising effects of the numerous peptides [25] that interact with GnRH at the level of the pituitary.

The model presented here that incorporates two pathways, designated Pathways I and II, which when conceptualised as an interaction between the Ca^2+^ and the cAMP/PKA pathways, neatly explains the augmentation of the LH response resulting from the activation of cAMP and enables the coherent interpretation of investigations into the role of cAMP in the LH response to GnRH. Extra understanding of interactions between cAMP and other systems is expected to further the model analysis. Recent molecular studies have concluded that interaction between Ca^2+^ and cAMP may be important [26–30]. Additionally, our results provide further evidence for a link between GnRH self-priming and biphasic responses to GnRH, which result from either pulsatile GnRH or continuous input. The model provides a foundation to describe the dynamic nature of the LH response, the understanding of which is central to efficient clinical modulation of fertility [31, 32].

In early investigations of the mechanism(s) of the LH response to GnRH, cAMP was investigated as an obvious candidate. Many of the attempts which sought to establish its role resulted in negative conclusions [33–36]. Some groups detected both a rise in cAMP and an increased level of LH secretion but observed that there was a lack of temporal parallelism and thence concluded that there was no direct link between the two events [37–40]. Our model incorporates features for which LH is acutely released prior to the full activation of Pathway II (which we conceptualised as involving cAMP).

The self-priming response to GnRH, but not the initial response, involved protein synthesis [8, 16, 41, 42]. Perfusion with cAMP analogues or forskolin of anterior pituitaries at pro-oestrus increased the LH response to GnRH after 30 min, imitating self-priming and requiring protein synthesis [8, 43]. It was suggested that cAMP is not involved in the acute release. This second phase of the response, but not the early phase, was inhibited by cycloheximide. It was concluded that cAMP stimulates protein synthesis and cAMP affects the second phase via an indirect process [44, 45].

Hence, it is consistent that controversy regarding the role of cAMP in modulation of LH secretion can be considered to be *partly a result of the effect on LH secretion being delayed, and the effect altering as the time from initial GnRH receptor occupancy proceeds.* In other words, the rapid and readily observed effect of the Ca^2+^-mediated pathway in the response of a gonadotroph to GnRH has disguised the delayed roles of cAMP by which it modulatesphysiological functions. The study investigated the likelihood of two interacting pathways being at the basis of both the shoulder on the LH surges and self-priming, and the model illustrates that this appears to be highly likely.
